# Roundtable on Deregistration and Gender Law Reform Internationally

**DOI:** 10.1007/s10691-022-09512-7

**Published:** 2023-03-23

**Authors:** Jess Smith, Pieter Cannoot, Pierre Cloutier de Repentigny, Lena Holzer, Shelley Leung, Tanya Ni Mhuirthile, Evan Vipond, Nipuna Varman

**Affiliations:** 1 Lincoln Law School, College of Social Science, University of Lincoln, Brayford Pool, Lincoln, Lincolnshire, LN6 7TS UK; 2 Faculty of Law and Criminology, Ghent University, Ghent, Belgium; 3 Department of Law and Legal Studies, Carleton University, Ottawa, Canada; 4 Department of Law, Goldsmiths, London, UK; 5 Dickson Poon School of Law, King’s College London, London, UK; 6 School of Law and Government, Dublin City University, Dublin, Ireland; 7 York University, Toronto, Canada; 8 Erasmus University Rotterdam, Rotterdam, The Netherlands

**Keywords:** Decertification, Gender, Law reform, Registration, Sex, State

## Abstract

In this roundtable discussion, early-career researchers working in the field of law, gender, and sexuality discuss international and trans-national developments to legal gender. ‘The Future of Legal Gender’ research project focused on the legislative framework of England and Wales to develop a prototype for decertification. The domestic legislation, however, was situated within a wider international context throughout the project. This roundtable discussion, therefore, provided an opportunity for reflection on the transnational issues raised by decertification, with a particular focus on developments arising in the jurisdiction(s) studied by the early career researchers. The roundtable began with a brief outline of these recent developments before moving to an open discussion on key themes including the value of reform on wider society, changes on-the-ground by non-state actors, and alternative processes for tackling gender inequalities without certifying legal gender. The online conversation took place on 28 June 2021 and has been transcribed and edited for continuity, clarity, and referencing.

## Jess Smith (Chair)

Let’s begin by discussing some of the international developments around decertification. Is the government in the jurisdiction of your research currently considering removing legal gender status or otherwise reducing its significance?

## Tanya Ni Mhuirthile

In Ireland there’s a dissonance between what the political part of government will do and what the civil service will do. There does seem to be some political will to explore being more open about registration. It’s been acknowledged that there are human rights interests at stake, and there’s a wish to vindicate them and to realise them. Specifically, there’s a political will to explore the idea of bringing in recognition for non-binary identities, and there’s a question remaining about what that would look like. But, at the civil service level, it seems the computer won’t allow it. There’s a feeling that we would have to overhaul *every* computer system and *every* administrative system throughout the service before they would look at making changes to registration.

In the review for the Gender Recognition Act 2015 (submitted in June 2018), there was a commitment to consider introducing a model of non-binary recognition. I was part of the review and the civil servants in the group wanted it to say that we all agreed in principle for non-binary recognition—that was unanimous—but this root-and-branch way of looking at systems across the civil service *would* have to happen before introducing non-binary recognition. I got the wording changed to “*may* happen”. I felt that was a huge win: there would be a possibility of introducing something and we could worry about computer systems afterwards.

## Jess Smith

Thanks, Tanya. These are important points which reflect a broader concern with how conversations around decertification tend to fall back on the practicalities of abolishing legal gender *now* as opposed to considering it as a future-oriented and imagined law reform. Our project is exploring decertification as a prefigurative law reform and, drawing from utopian lines of thought, posing a question about decertification which is positioned outside of the restraints of the political ‘now’ (Cooper 2020). A wider conversation around legal gender tends to come back to what’s practical (and desirable) in the current political climate or suggests pluralising legal genders as a stepping-stone to wider reform. If reform is being proposed, how often is this approached as a question of adding gender categories rather than dismantling the system of gender registration?

## Pieter Cannoot

In 2019, the constitutional court of Belgium found parts of our Gender Recognition Act, which was reformed in 2017, to be discriminatory and therefore unconstitutional, because of the lack of recognition of non-binary persons. The Belgian judgment was similar to decisions made by the German and Austrian constitutional courts: the issue of discrimination could be resolved by either adding one or more [gender] categories to registration—both at birth and in gender recognition procedures—or to abolish sex/gender as an element of legal personhood.^1^ And because Belgium has had a long government crisis, which was only solved because of the Covid-19 pandemic, we are currently still in the phase where the government is inviting advice from scholars. The current federal government, which is now composed of progressive parties, are willing to look beyond adding one or more categories for gender recognition.

## Shelley Leung

In Hong Kong, same-sex marriage is not legal. In the 2013 Court of Final Appeal case *W v Registrar of Marriages*,^2^ a trans woman sought permission to marry her male partner. In Hong Kong, an individual must undergo gender affirmation surgery to be legally recognised in their desired gender. W had completed the surgery, but the issue was whether marriage was uniquely between a man and woman. If the court recognised W as a woman, then they would be recognising the legal right of trans individuals to marry, but if they didn’t recognise W as a woman then the judgment would have the effect of recognising same-sex marriage. The Court of Final Appeal decided that W had the right to marriage. The decision led to an inter-government departmental working group being set up to examine legal gender recognition in Hong Kong. In 2017, the interdepartmental group issued a public consultation paper asking different organisations as well as the public to submit their views about legal gender recognition (Inter-departmental Working Group on Gender Recognition 2017). Many of the opinions submitted to the group were negative, indicating that the wider public were not very receptive to changing laws concerning legal gender recognition. There was supposed to be a second round of consultation, but because of the current political atmosphere in Hong Kong and the ongoing pandemic, legal gender recognition is not at the top of the political agenda. So, right now the government doesn’t have any incentive to introduce legal gender recognition laws or to look at reducing the significance of legal markers.

## Nipuna Varman

In 2014, the Supreme Court of India officially recognised a third gender, saying that trans people are now the third gender, which is the ‘transgender/third gender’ option that’s on forms you fill in when you are in India.^3^ The Transgender Persons (Protection of Rights) Act 2019 was introduced following the Supreme Court judgment. The legislation defines trans persons as trans men and trans women along with intersex people, genderqueer people, and people who identify themselves under socio-cultural categories such as hijras, jogtas, and kinnars. Although the Act defines trans persons in this wider sense, it goes back to the medical model and says that if a trans person wants official documentation to recognise their identity as a man or woman, then the individual will have to undergo medical procedures and gender forming surgeries.^4^ The individual then has to go before the district magistrate in the lower court and a legal official has the power to determine whether you will get that certification or not.

No one is talking about decertification, as such, but there is discussion in India around gender, and a wider movement which discusses removing caste and religion from the birth certificate. There are cases pending in the lower courts right now which fight for such a thing. Unlike a lot of Western phenomena, where race and gender are seen as intersectional, in India, caste and gender is co-constitutive. The patriarchal order feeds into caste and gender hierarchies. So, when we talk about caste, we talk about gender and when we talk about gender we talk about caste. So, I feel that if there’s discussion around removing caste from identity documents, there is scope to talk about gender as well. I am currently researching how decertification would affect caste identities, particularly members of castes who face oppression, and it’s important to note that when you talk about decertification in India, you also need to then think about it in terms of how it effects caste identity.

## Pierre Cloutier de Repentigny

Canada has the federal government, plus each of the provinces and the three territories, and in some cases, Indigenous nations all have their own regulations and law regarding gender, so it can be hard to keep track of who is doing what, at a national level. But, right now, I think there’s no major push, at least from the government’s perspective, at completely removing gender. Most governments in Canada are starting to add extra categories. Ontario was one of the first to do it by using the ‘X’ mark as a third gender, additional to the binary system, and introducing the option of not having your gender displayed at all on certain government documents, like birth certificates.

There is an interesting judgment delivered, in February 2021, by the Quebec Superior Court which challenged the Civil Code of Quebec and the provisions that mandate the recognition of gender.^5^ While the judge recognised that assigning a gender at birth discriminated against transgender and non-binary people, he found this discrimination justifiable on the basis that this designation could be changed later in life and on the alleged importance of the designation for the provision of public services. Justice Moore found that this purpose of sex designation outweighs the violations of gender non-conforming people’s dignity and equality. It’s judicial interpretation, but in Canada, changes in the law concerning gender have tended to come from judicial decisions, and often based on human rights legislation or the constitution. The case was first instance, so we might see a change once the Court of Appeal renders its judgment, but that’s one of the most recent developments in Canada.

## Evan Vipond

One of the biggest concerns or arguments against removing the gender marker on passports (a federal document that has the option of an X gender marker) is that of the challenge of international travel. The Government of Canada warns citizens that having an X gender marker could pose difficulties at certain international borders that don’t recognise a third sex/gender category. At the federal level, then, there’s no push to move away or towards decertification in terms of those documents.

In Ontario, the province I reside in, while there isn’t a push for decertification, there is a shift towards reducing its significance in certain instances. For example, sex designation is no longer visible on provincial health cards. A lot of people might think that means one’s sex designation is no longer tied to their health information and identification but within the computer system, a binary sex designation remains in place—it doesn’t just vanish.

Also, with the Ontario driver’s licence, there’s also been an addition of the X marker, but at least in Ontario, X does not represent non-binary gender identity or third sex, it indicates an ‘unspecified’ sex designation. If you get an X on your driver’s licence, an M or F remains in the system, it’s just not visible on your card. This means that to get the X, you don’t have to necessarily prove that you’ve transitioned, because it’s not actually a third sex/gender designation. It’s more akin to opting out of a binary sex category. I know some jurisdictions have sort of created the X to say, this is a third sex/gender category, and there’s a public assumption that the X category functions like that in Ontario.^6^ Some trans people might be disappointed to find out that X doesn’t actually indicate a non-binary gender identity or third sex designation; rather, it is a catch-all for trans, non-binary, Two Spirit, and binary people who don’t want to disclose their gender identity, without being restricted to trans, non-binary or Two Spirit people. The fact that there is this sort of unspecified category is a small step towards reducing perhaps the significance of legal gender because it’s essentially saying okay, you don’t have to necessarily have a sex marker that’s present. So, the ‘X’ model appears to be more progressive than it is or perhaps appears to be a non-binary and open model of registration. But it’s not necessarily the case.

## Lena Holzer

In March 2021, the cantonal court of Aargau recognised that a Swiss National erased their legal gender in Germany. It held that according to Swiss private international law, the principle of public policy cannot be invoked to deny the recognition of a foreign legal gender status, such as the status of being legally non-gendered.^7^ I would assume the relevant public institution for civil registration of Aargau is going to appeal the case, because of the issues around data management that Tanya was discussing in the Irish context, since Switzerland has a centralised registry system, which has currently only the technical capacity to register an F or M. In Switzerland, information about an individual’s legal personhood is registered in one central registry, which is a bit different from other federalist states, such as Canada, where different documents can show different gender markers. Yet, the Court of Aargau held that the technical limitations of not being able to record a category other than F or M by the Swiss electronic registration system cannot justify refusing the recognition of a foreign legal gender status. As a caveat, however, the case is limited to the point of recognising the status of being formally non-gendered that was acquired in another country. Hence, the case does not grant Swiss Nations the right to erase their legal gender in the Swiss civil registry.

## Evan Vipond

I just want to quickly add that, in the context of Canada, one of the things I foresee that could similarly be a concern with decertification is around the Indian Act, which only applies to indigenous people, and distinguishes between indigenous men and women. If there was a change for decertification then it would have to consider the question, does that extend to the Indian Act or indigenous people, and what would that mean?

## Lena Holzer

I wanted to ask Nipuna whether some castes are currently noted on identity documents and, if so, whether they are noted for everybody?

## Nipuna Varman

It’s in the sense that you get these certificates, but it’s a little complicated. When the constitution was put in place in India, there was also this concept of scheduled caste and scheduled tribes that list out certain castes and tribes who are oppressed communities in India. And these communities are given benefits to affirmative action policies and all those things. If you want to avail such policies, you need to have a certificate that says that you fall within the community that’s under the schedule. And that’s sort of how you go around it. In educational institutions, when you apply to colleges, there are lists that are put out for people who belong to such communities and the rest come under this list called the general category—and these are the people who do not fall within the category of oppressed communities.

## Jess Smith

Thank you, everyone. Can we now think about the value of decertification and what that might do in law and society that some of the other options (such as multiplying gender categories or using an ‘X’ marker) may not? What are the political and policy benefits that might follow from the state’s withdrawal from certifying legal gender?

## Pierre Cloutier de Repentigny

In Canada, there is a resurgence of Indigenous sovereignty and self-determination which includes putting at the forefront different gender identities that are not congruent with Western ideas of gender identity. In states where you’re trying to regulate gender, especially the binary way, but even if you’re trying to move around in a non-binary way, by just adding a generic category of ‘other’, this has implications for recognising differences that are often subsumed in the category of Two Spirit. But that’s in and of itself a category that considers multiple identities and is a recent terminology. So, I think there is some benefit, from an anti-colonial perspective, to trying to remove the state away from regulating gender.

Kim TallBear (2020) has discussed how, from a sexuality point of view, (but I think it applies equally to gender identity), the state has tried to regulate gender, marriage, and sexuality to control Indigenous populations by trying to destroy the qualities that make people Indigenous and trying to impose a certain view of the Western state on them. This permeates outside because it’s also a way of controlling the non-Indigenous population because it’s trying to fit everyone neatly into these distinct boxes that are more easily controllable and categorisable and therefore a state can impose legal obligations or duties differently based on that. The moment you try to add a new category it becomes complicated so it’s not in the interests of the state to recognise multiple genders.

It’s the idea of just adding one more. But even that becomes complicated because many jurisdictions have gendered language, I think Quebec being one of the most prominent examples in Canada. It’s an issue of French, compared to English, because in most English jurisdictions, the law becomes gender-neutral so you have ‘spouse’ instead of ‘husband’ or ‘wife’, ‘person’ instead of ‘man’/‘woman’. But Quebec still retains a lot of the language of mother/father. The state simply recognises these categories as imperative and fits people into them. If you give birth regardless of your gender, in the eyes of the state, you’re the mother.^8^

At least trying to step back from that would remove these kinds of legal fiction that are often complicated and hurtful from the point of view of the individual that must deal with them. People who view themselves as mother are being fit in the category of father by nature of not having given birth or let’s say you have two lesbian parents, one will be the father regardless because of how the system is created. It’s partially because rights are assigned differently in that, for example, parental leave is lesser for the father than it’s for the mother, instead of leaving it to families to decide how to approach it.

The final question is to understand from a power relation perspective as to *why* the state regulates gender categories. What is the purpose of this regulation? And I think decertification, regardless of whether it’s achieved or not, if pushed, at least I think brings to the forefront this idea of why states do this in the first place. In Canada, recent court cases, and especially in the one I mentioned in Quebec, have started to push question of why do we register gender? Why do we start from a point of differentiation [in legal gender categories] if we’re trying to move towards a policy where people would have similar obligations based on capacity rather than on identification like gender?

## Jess Smith

Thanks, Pierre, you raised important points there that reminded me of James Scott’s (1998) argument in *Seeing Like a State* about the imperial state and its categorising logic. Scott argues that the state produces a map of identity from which it can then partially see—in the sense that the state sees what it wants to, and that map forms the premise from which it acts. The contemporary discussion around decertification is also now highlighting the digitalisation of James Scott’s arguments in debates about what the computer sees and who controls the computer.

## Pieter Cannoot

I wanted to raise a couple of similar issues to those mentioned by Pierre. In Belgium, gender registration and reform are always until now conceptualised as a trans issue. It’s a debate that’s considered to concern trans persons and their suffering and the need to conduct a better situation that accommodates their needs. The decertification debate makes it clearer that gender affects *everybody* in society in that everybody from birth onward is gendered. Decertifying legal gender would potentially lead to a situation where the state can tackle some of the gender oppression that exists in societies. It’s only when we, at least in Belgium, started talking about the possibility of removing legal gender, that many people started to realise that they are gendered as well even when they do not necessarily have a problem with the gender marker or have never really considered the fact that they need to identify in a certain way. The value of debating decertification is that it helps people to realise that they are structurally gendered in society, even before they are born. It could also lead to removing these very intense forms of heteronormativity, particularly of course in family law. Decertification has the benefit of starting discussions about remaining heteronormative areas of law.

## Jess Smith

In England, when civil registration was first introduced in the Victorian era, it was never discussed whether gender should be recorded. The discussions were based around whether the baptismal name should be recorded and by whom because registration was being taken out of the control of the Anglican church to create a civil system (see for discussion, Szreter and Breckenridge 2012). So, I agree, more recent discussions about decertification are some of the first conversations around what has really been an assumed norm.

## Evan Vipond

Once there are more shifts to the language used in law and legislative frameworks, then it becomes increasingly less relevant, particularly in the sense we have so many human rights that don’t require legal proof of membership rights. You’re not legally designated into categories of race, ethnicity, sexuality, or class, but you still have legal protections.

In terms of the Indian Act, as I previously mentioned, it could also, in some ways, be beneficial because then they would have to redraft the Indian Act to be gender-neutral and therefore no longer patriarchal in the way that it was then. That would be beneficial because then Indigenous people in Canada would be treated the same regardless of gender. The existence of the Indian Act itself, of course, is contentious. But in terms of creating a gender-neutral document that would be a step towards, at least, decolonising sex and gender.

There are a lot of opportunities for decertification and the government has already started separating sex and gender to the point that it now begs the question, is sex registration relevant? If the government can recognise that you might be assigned one sex and have a different gender, then what is the value of that sex designation if you recognise gender differently? This year’s census in Canada, for instance, asked about your sex assigned at birth and asked about your gender identity. Whilst that doesn’t hold any weight in terms of the law at this moment, it does show that the government is demonstrating an understanding that perhaps there’s a difference between sex and gender and that a lot of our laws are based more on gender assumptions, so is this necessary? Is the state’s designation of sex necessary if we already recognising that people have various gender identities?

## Jess Smith

Thank you, that’s an important question that I’d like to come back to later. Nipuna, did you want to come in?

## Nipuna Varman

I don’t know how practical decertification would be in India, given the current situation, but it’s an important discussion to be had. During the pandemic, the state announced that money would be deposited in the banks of people who identify as trans.^9^ The census data has recorded about 40,000 people who recognise themselves as trans but because it’s so difficult to get your gender identity recognised by the state in your official documents, many of those who identity as trans do not have bank accounts with this information. Without official documentation, you are not able to access the benefits that the state bestows upon people. What is the importance of recording gender when, whatever the arguments you are making in favour of recognising gender legally, it’s not working on the ground?

## Jess Smith

Thank you, Nipuna. I wonder if we can continue your discussion by thinking more about legal pluralism and the reach of the state. What is the role of religious authorities, amongst other non-state actors, in the legal production of gender? Our project is exploring the ways in which decertification highlights how it’s not just a central state that is responsible for categorising sex and gender. Every day, decisions about gender are being made and unmade by unions, non-governmental organisations, and local councils, to name a few. Take for example, the growing discussion around pronouns and the different bodies that are introducing pronoun badges or signatures as a way of challenging, to some extent, normative assumptions about sex/gender. Do we need to register sex if gender is doing different conceptual work? Does the state need to record sex if other actors are doing and undoing that work? I wondered if anybody wanted to come in with reflections from their jurisdictions about different actors that have been involved in discussions about regulating sex/gender.

## Evan Vipond

At York University (Toronto), we have a strong union for grad students and contract faculty, and we have repeatedly petitioned the university to push back against their use of sex/gender markers within the administration. We’ve argued for the importance of using lived names, even if it’s not a student’s legal name. We’ve been able to get some movement there through the union which has pushed the university to not necessarily designate people based on their legal status. That’s one example of where an institution is moving away from relying on state designation, whether it’s in the case of names or a gender marker.

## Tanya Ni Mhuirthile

In Ireland, many of the older schools in the country are gender segregated. When we became our own state 100 years ago, the newly formed state handed over all responsibility for healthcare and education to religious organisations. Some of the ones in the countryside where numbers are small would be integrated, but a lot of schools in Ireland are gender segregated. Most pupils would wear uniforms in school, and there will be precise dress codes which are gendered. It’s only been in the last six or seven years that I’m seeing girls in girl’s schools wearing trousers, for example, as opposed to being mandated that they must wear skirts. A trouser option is new. These dress codes can cause untold stress for pupils. Some schools will be flexible but dress codes are administered on a school-by-school basis because there is no central authority coming from the government.

In the universities here, if you want to be known by a name other than that legally documented, perhaps to better reflect an individual’s gender, the universities have what they call a nickname situation. It was originally introduced for Asian students who wanted to have western sounding names. The chosen nickname goes on identity cards (ID cards) and email addresses, but the degree has the legal name on it. So, there’s a work around for everyday life and administration within the university, but formal legal records will have your formal legal name on it.

## Jess Smith

It seems that one of the challenges to be addressed when thinking about decertification as a tool to dismantle broader structures of sex/gender is the formalisation that law is so steeped in, whether it’s the formality of a dress code or a legal certificate. If decertification is to do the work of challenging gender norms, do we need to see a broader de-formalisation of societal values and systems alongside the stepping back of the state? Would decentralising legal gender create ad hoc policies, and if so, would a situation-by-situation approach to regulating sex/gender further entrench gender inequalities and oppression if there’s not a legal standard to steer decision-making by non-state actors or could it allow for more flexibility?

## Tanya Ni Mhuirthile

In Ireland, there’s a view amongst secondary schools that flexibility to make decisions around sex/gender has been replaced by the statutory gender recognition process. The flexibility has disappeared because there’s a legal norm that must be adhered to.

## Pieter Cannoot

I want to return to Evan’s question—is the state’s designation of sex necessary if we already recognise that people have various gender identities? There seems to be a push right now to separate sex and gender registration, if any form of registration is maintained, and to move sex registration to a purely medical register. In Belgium, every newborn receives an E-patient file. Some people have been asking the government to include sex registration in that E-patient file, and not just having male or female categories but, working with criteria, recording data that might have an influence later. There could be a spectrum or continuum for recording sex at birth and if gender registration was maintained it would be a separate database, and only registered from, let’s say, when the individual reaches 12 onwards because before that age, the person can’t know or express their gender in a way which would satisfy what the law would require from the registration.

To provide an example, it’s useful to know data on boys’ and girls’ education for the purposes of achieving gender equality, so there’s a suggestion of registering gender at enrolment and communicating it to the government. Gender data from enrolment would then inform public policy [which aims to challenge gender stereotypes] by getting more female students into maths or science subjects and male students in caring or nursing professions, etc.

## Jess Smith

Thanks, Pieter, that’s an important point. Is it possible to record sex/gender in aggregate for policy reasons, for example, collecting data about boys’ and girls’ schools to reflect on what might be educational differences? Could a progressive state detach recording sex/gender at an individual level, where it becomes a facet of legal personhood, from recording it at aggregate level, so the state could continue tackling gender inequality and oppression?

## Lena Holzer

In Switzerland, sports federations make the decision [who can play in gender-segregated competitions] often on a case-by-case basis which creates a lot of insecurity for players. The decision of the federations depends on different factors. Sometimes the legal gender will be relevant but often it will be replaced by a metric such as the level of testosterone which is circulating in your body. Sometimes access to sex-segregated sports is based on self-declaration. It often depends on the person who is making the decision, and on the sport. At international level, there are some very restrictive regulations, which makes it difficult for trans and intersex amateurs and professionals to play in the categories they want to compete in. However, the lack of regulations or guidance from international sport federations can put players at risk of being treated arbitrarily based on the understanding of an official who administers the national club teams.

There are parallels in how international sports bodies and the state regulate membership to legal sex/gender categories. There’s always an interplay between stepping back from the state and using the state’s regulation as a protective mechanism. Without state guidelines on regulating sex/gender, the individual can be rendered vulnerable to arbitrary judgment (for example, by a school or basketball club). But, at the same time, the state’s regulation may reduce flexibility such that schools don’t want to use the model of self-declaration anymore because they become fixated on the Gender Recognition Act and require all individuals to go through the state-governed gender recognition procedure before recognising them in their gender identity. It’s a dual movement of reducing state’s power to classify individuals into gender categories, while potentially increasing the power of non-state actors to make decisions on gender classifications.

## Shelley Leung

I want to talk about access to facilities and public education in Hong Kong. The question of decertification is a bit more complicated in Hong Kong because children born in Hong Kong are issued a birth certificate which records a legal sex marker, and a Hong Kong ID card, that is used to access public facilities and verify identification. We use that card for everything. If an individual wanted to buy concert tickets, for example, the seller would typically ask for your ID card number and other details listed there, such as name, legal sex marker, and date of birth. To continue the example of education, the best schools in Hong Kong are usually sex segregated. These schools rely on guidance from the Hong Kong Education Bureau and when they decide whether a child can be admitted to say, a sex segregated school, they assess according to the marker on the ID card. The ID card underpins so much of everyday life in Hong Kong that it would be a challenge to implement decertification, given how things currently stand.

## Pierre Cloutier de Repentigny

I think parts of the state when they’re not necessarily attached to a legal obligation tend to be significantly more flexible than how the registration of gender generally operates. Take for example, affirmative action or equity programmes where gender becomes embodied in its multiplicity. The language of gender has now become, in terms of policy and state programming, all-encompassing, it’s basically not cis male. [In 2018], the federal government changed the name of the ‘Status of Women Canada’ department to the ‘Women and Gender Equality Canada’ [which is a more expansive use of gendered language]. The re-naming also subsumes sexuality into it, as the department deals with the sexual orientation policy issues of the government. Gender has become a broad and flexible category which is starting to permeate a bit everywhere in terms of civil society.

How does the state avoid a model of decertification which removes itself completely from intervening in matters of gender? How does it balance the need to intervene on equality grounds and protect people who are vulnerable, based on structural power dynamics, without doing the work of administering gender categories and adopting a model of gender recognition where it’s a fixed component of legal personhood? These are questions which reveal a fragmentation in progressive discourse. There’s no singular conservative or progressive approach to shifting towards different understandings of gender or tackling gender oppression.

## Lena Holzer

If the state decertifies gender, I think other bodies, such as sports federations and clubs, will continue to validate gender. The question then becomes, what’s the role of the state in the validation process conducted by private bodies?^10^ We would have to consider what we want the role of the state to be in conditions of decertification because systems of validation will continue to work at different levels and spheres—as they are working now.

## Jess Smith

Yes, and to continue the discussion, it’s worth asking how could the state tackle gender inequalities and other structural issues without assigning or policing membership of categories?

## Nipuna Varman

These are complex questions as the best way to achieve gender equality without using gender classifications will change based on geographical and cultural context. Gender inequalities often stem from social negotiations rather than legal status. Take for example, a temple in India that prevents women of reproductive age from entering and offering prayers in the temple. The religious organisation makes the distinction of who is a woman (or who is a man) on the gender the person *appears* to perform rather than the legal gender the person carries. I believe that legal gender, in that sense, does not provide an avenue for people seeking justice.

## Pieter Cannoot

Decertification of legal gender is not a goal on itself. It is an important tool in the broader struggle to end gender inequalities in society. We can never talk about decertifying gender, without talking about gender justice. Self-determination of gender is not limited to questioning the conditions of legal gender recognition and discussing the boxes available. It is more broadly concerned with negative and positive state obligations which allow all people to be free from gender harm and stereotypes. There is a risk, otherwise, that gender is seen as a matter of personal identity, or even personal choice, which in a liberal state does not concern the state. Yet, gender is also a cultural system that produces and reproduces gender inequality and disadvantage.

We must resist the idea that there will be an all-encompassing solution to deal with gender from a legal point of view. The role of legal gender in protecting trans persons against discrimination in employment might differ from its role in supporting persons affected by domestic violence in shelters and government programmes. However, being sensitive to the different roles that genders play, does not mean that gender identity needs to be registered by the state, or that there needs to be an official definition of what gender is.

## Lena Holzer

Feminist organisations in Switzerland have long pointed out that systematic underpay and undervaluing of care work, lack of affordable childcare, insufficient parental leave, and the taxing system (which often encourages women in heterosexual marriages to give up paid employment) strongly contribute to women’s lower socio-economic status in relation to men in Switzerland. Gender inequalities could be tackled by changing labour and economic laws and policies that foster the hierarchical gender division of labour.

## Evan Vipond

I don’t think gender inequalities (and structural dynamics) can be addressed through state-certified identity categories. In the case of Canadian law, and most Western states, the law was founded on principles of patriarchy and white supremacy, and was designed to privilege white, cis-heterosexual, nondisabled, property-owning males. While women have achieved legal equality in Canada, it has not resulted in social, political, or economic equality. I am sceptical, therefore, that continuing to put people in ‘boxes’ (even if the number of boxes was expanded to include additional identity categories) would tackle gender inequality. Certification (re)produces gender inequalities such as the subordination of women, inequalities between women, and between cisgender and transgender persons. The burden of having to legally change one's sex/gender marker is placed exclusively on trans, nonbinary, Two Spirit, and intersex individuals—having additional gender markers would not remove this burden. It would be far more beneficial for those in need if we focused our attention on how oppressive systems are structured and operate, rather than how people are categorised within them.

We need to address the effects of harms that have been caused, rather than predetermine who can experience harm based on legal categorisation.

## Footnotes

1 Bundesverfassungsgericht 10 October 2017, 1 BVR 2019/16 (Germany); Verfassungsgerichtshof 15 June 2018, G 77/2018-9 (Austria).

2 [2013] HKCFA 39.

3 *National Legal Services Authority v Union of India* and the citation for the same is Writ Petition (civil) No. 604 of 2013.

4 The Transgender Persons (Protection of Rights) Rules, 2020 (‘Rules’) undoes the Act slightly by defining medical intervention widely (such intervention would include counselling, hormonal therapy, etc.). The Act used the term ‘surgery’ but the Rules have a wider construction.

5 Centre for Gender Advocacy c. Attorney General of Quebec, 2021 QCCS 191 (CanLII).

6 Government of Ontario. 2016. Gender on Health Cards and Driver’s Licenses, Government of Ontario. https://news.ontario.ca/en/backgrounder/40957/gender-on-health-cards-and-drivers-licences. Accessed 4 April 2022.

7 Obergericht Kanton Aargau, 29 March 2021.

8 See for case law in England and Wales, *R (on the application of McConnell) v Registrar General* [2020] EWCA Civ 559.

9 In May 2021, the Ministry of Social Justice and Empowerment announced that transgender persons in India could apply for a one-off payment of 1500 rupees recognising “serious disruption of livelihoods” due to the COVID-19 pandemic. Applicants had to submit basic details, Aadhar (unique identity number) and bank account number via an online form. See Ministry of Social Justice and Empowerment (2021).

10 See for example, *Semenya v Switzerland* (pending) European Court of Human Rights Application 10,934/21.

## References

[CR1] Cooper Davina (2020). Towards an Adventurous Institutional Politics: The Prefigurative ‘as if’ and the Reposing of what’s Real. The Sociological Review.

[CR2] Government of Ontario. 2016. Gender on Health Cards and Driver’s Licenses. https://news.ontario.ca/en/backgrounder/40957/gender-on-health-cards-and-drivers-licences. Accessed 28 September 2022.

[CR3] Inter-departmental Working Group on Gender Recognition. 2017. Consultation Paper: Part 1 Gender Recognition. https://www.iwggr.gov.hk/eng/pdf/consultation01.pdf. Accessed 28 September 2022.

[CR4] Ministry of Social Justice and Empowerment. 2021. Government to Give Assistance of Rs.1500 to each Transgender Person in view of Covid Pandemic. https://www.pib.gov.in/PressReleasePage.aspx?PRID=1721277. Accessed 28 September 2022.

[CR5] Review Group of the Gender Recognition Act. 2018. Review of the Gender Recognition Act 2015: Report to the Minister for Employment Affairs and Social Protection. https://www.gov.ie/en/press-release/6b7814-review-of-the-gender-recognition-act-2015-report-to-the-minister-for/. Accessed 28 September 2022.

[CR30] Scott, James C. 1998. *Seeing Like a State: How Certain Schemes to Improve the Human Condition Have Failed.* New Haven; CT: Yale University Press.

[CR6] Szreter, Simon, and Keith Breckenridge. 2012. *Registration and Recognition: Documenting the Person in World History*. Oxford: The British Academy by Oxford University Press.

[CR7] TallBear Kim, Hokowhitu Brendan (2020). Identity is a Poor Substitute for Relating: Genetic Ancestry, Critical Polyamory, Property, and Relations. Routledge Handbook of Critical Indigenous Studies.

